# Characterization of Hybrid FRP Composite Produced from Recycled PET and CFRP

**DOI:** 10.3390/polym15132946

**Published:** 2023-07-04

**Authors:** Ghdayra Almahri, Kaouthar Madi, Fatima Alkaabi, Yahia Badran, Khaled Shehadeh, Amged ElHassan, Waleed Ahmed, Salem Alzahmi

**Affiliations:** 1 Department of Chemical & Petroleum Engineering, United Arab Emirates University, Al Ain P.O. Box 15551, United Arab Emirates; 201803824@uaeu.ac.ae; 2 Department of Mechanical and Aerospace Engineering, United Arab Emirates University, Al Ain P.O. Box 15551, United Arab Emirates; 201735186@uaeu.ac.ae (K.M.); 201700795@uaeu.ac.ae (F.A.); 202050911@uaeu.ac.ae (Y.B.); 202050932@uaeu.ac.ae (K.S.); 201450104@uaeu.ac.ae (A.E.); 3 Engineering Requirements Unit, United Arab Emirates University, Al Ain P.O. Box 15551, United Arab Emirates; w.ahmed@uaeu.ac.ae; 4 National Water and Energy Center, United Arab Emirates University, Al Ain P.O. Box 15551, United Arab Emirates

**Keywords:** recycling, PET, composite, properties, CFRP

## Abstract

In recent years, carbon fiber has experienced a significant surge in popularity attributed to its exceptional properties, including its high-temperature resistance, mechanical strength, and cost-effectiveness. Many industries have been attracted to the prevalent use of carbon-fiber-reinforced polymers or plastics (CFRP). However, the increasing demand for carbon fiber has created a waste recycling problem that needs to be addressed. This research aimed to develop a recycled composite using PET waste as a solution to the growing demand for both materials. The recycled carbon fibers were processed chemically and mechanically to generate power for this process. Various samples were tested with different proportions of CF (10%, 20%, 30%, and 40%) to analyze their mechanical properties. The recycled composites are examined under tensile test conditions to further explore the waste carbon reinforcement’s effect on polymers’ characteristics. Scanning electron microscopy was also utilized for mechanical morphology evaluations. After analyzing the data, it was found that samples containing 20% CF had the highest elastic modulus value among all the mixes. This is attributed to the reinforcing effect of the fibers. The Elasticity Modulus of the filaments increased with the concentration of CF, reaching its peak at 20% before decreasing. This trend is also apparent in the visual representations. When compared to recycling, the Elasticity Modulus value of 20% CF filament increased by 97.5%. The precise value for CF with a 20% filament is 4719.3 MPa. Moreover, the composite samples were analyzed using SEM to characterize them, and it was discovered that the incorporation of 20% CF/PET filler produced the composition with the highest strength.

## 1. Introduction

Carbon fiber, also known as “graphite fiber” [1], possesses a unique combination of high strength and lightweight properties, making it an ideal material for manufacturing airplane components, particularly when used in the form of Carbon Fiber-Reinforced Polymer (CFRP) composites. Three methods are available for chemical recycling: conversion, depolymerization, and dissolution [2,3]. In comparison, mechanical recycling, which is the most commonly used method for recycling plastic, is suitable for thermoplastics such as PET. These polymers can be melted and solidified repeatedly without altering their chemical structure [4]. Mechanical recycling involves several steps. The first step is to collect the plastic that can be recycled. The second step is to separate the plastic from other materials, which can be achieved manually or mechanically [5]. The third step is to thoroughly clean the plastic to avoid affecting the quality of the final product. The plastic is ground and shredded into smaller pieces in the fourth stage. The last step is to melt the plastic and extrude it into pellets [6]. Dissolution follows a similar process to mechanical recycling in the initial stages, which includes collecting, sorting, and preparing the plastic. Subsequently, solvents and heat are employed to divide the plastic into a solution of polymers and additives, which is later retrieved. The additives are subsequently removed, and the polymers are added to create recycled plastic [7]. Depolymerization, also known as solvolysis or chemolysis, is the process of breaking down polymers into monomers, which are the building blocks of polymers. This is accomplished through various chemical reactions, solvents, and heat. In the initial stages, the plastic to be recycled is processed and prepared, and impurities are removed. The resulting monomers are then used as a secondary raw material in traditional plastic production [8]. To ensure the production of high-quality products, the conversion recycling process involves preparing and screening plastic waste, then converting it into a raw material or feedstock that takes the form of gas (gasification) or oil (pyrolysis), which can be utilized to generate chemicals in the future. To ensure the production of high-quality products, the gasification process is carried out in the presence of oxygen instead of pyrolysis, which takes place in the absence of oxygen. Any potential impurities in the resulting gaseous or oil feedstock are then removed so that they can be re-integrated into the chemical manufacturing chain and yield products such as polymers [9]. Carbon fiber is popular in various industries due to its unique properties, including its resistance to high temperatures, mechanical strength, and reasonable cost. Here’s how these properties contribute to its popularity. Carbon fiber exhibits excellent heat resistance, allowing it to maintain its structural integrity even at high temperatures. This property makes it suitable for applications in the aerospace, automotive, and energy industries, where components may be exposed to extreme heat. Carbon fiber is incredibly strong and rigid, providing a high strength-to-weight ratio compared to traditional materials like steel. This property makes it desirable in industries where lightweight yet strong materials are required, such as aerospace, automotive, sports equipment, and construction. Over the years, the cost of carbon fiber production has been reduced, making it more economically viable for various industries. While carbon fiber is still relatively more expensive than conventional materials, its benefits in terms of strength and weight reduction often outweigh the higher upfront cost, especially in high-performance applications. However, the growing demand for carbon fiber also presents challenges in waste management and recycling. Carbon fiber composites are challenging to recycle due to their complex structure. The carbon fiber is typically combined with resins or other materials, making it difficult to separate and recover the fibers effectively. The recycling process requires specialized techniques and technologies to break down the composite and extract reusable carbon fibers. Recycling infrastructure for carbon fiber is still relatively limited compared to other materials. The lack of widespread facilities and expertise in carbon fiber recycling hinders efficient recycling practices. This leads to a significant portion of carbon fiber waste ending up in landfills or incinerators instead of being recycled. Carbon fiber production involves energy-intensive processes, including the use of fossil fuels and chemical treatments. Improper waste management and disposal of carbon fiber can contribute to environmental pollution and greenhouse gas emissions. Therefore, developing effective recycling methods is crucial to reducing the environmental impact of carbon fiber waste. Efforts are being made to address these challenges and improve carbon fiber recycling techniques. Researchers and industry professionals are exploring innovative methods such as pyrolysis, solvolysis, and advanced mechanical recycling to enhance the efficiency of carbon fiber recycling and reduce waste. Additionally, promoting awareness and investing in recycling infrastructure are essential steps toward the sustainable management of carbon fiber waste [10].

Polymer matrix composites have made a name for themselves in the materials world due to increased features such as high specific stiffness and strength, durability, and so on. The matrix material in a variety of polymer matrix composites is recycled polyethylene terephthalate (RPET). Composite materials based on the RPET matrix are not only cost-effective but also environmentally beneficial [11]. The volume ratios of composite materials reinforced with waste paper and matrix consisting of epoxy resin or hybrid resin at 60%, 70%, and 80% Dammar were investigated. Tensile testing and scanning electron microscopy (SEM) analyses were performed on all samples collected. The tensile response, tensile strength, modulus of elasticity, elongation at break, and fracture surface analysis were all determined [12].

Waste management and recycling companies are developing innovative technologies and procedures to recycle carbon fiber waste in order to meet these difficulties. Recycling technological advancements such as pyrolysis, solvolysis, and thermolysis are making carbon fiber recycling more viable and cost-effective. Furthermore, some companies are using closed-loop manufacturing procedures, which allow for the reuse of carbon fiber waste generated during the manufacturing process.

Pyrolysis and solvolysis are the most developed and prosperous chemical processes for recovering carbon fibers. Pyrolysis is widely used in industry due to its low cost and ease of use [13]. Pyrolysis is a thermochemical process that breaks down the organic component of composite materials in the absence of oxygen, typically between temperatures of 450 °C and 700 °C. This process involves two main steps: the thermolysis/pyrolysis step and the gasification/oxidation step. In the first phase of the process, the material is heated to high temperatures in a furnace to separate the fibers from the organic matrix (often resin). At the last step of the process, the char deposit on the surface of the fiber is removed using airflow. These steps aim to preserve most of the original properties of the fiber [14]. Nevertheless, it has been reported that this recycling method generates composites with inconsistent qualities that are not as good as those made from pure carbon fibers [15]. Therefore, the recycled product obtained through this method cannot perform the same functions as virgin carbon fibers. The outcomes of their study demonstrated that the carbon fibers maintained their elastic modulus and retained 90% of their tensile strength compared to virgin fibers under ideal conditions (thermolysis process at 500 °C for 6 h, followed by a gasification step in the air at the same temperature for 30 min) [16,17,18,19]. In 2018, Boeing and ELG collaborated to use pyrolysis to recycle their CFRP, resulting in aerospace-grade composite material used to manufacture items such as laptop cases and car parts, reducing annual solid waste generation by nearly one million pounds [20].

In recent years, fiber-reinforced composites have gained attention as a possible substitute for metal parts in various industries due to their superior performance under fatigue conditions, lower density, higher specific strength, and stiffness compared to conventional metals [20,21]. Structural component design should consider the behavior of these composites under different loading conditions, including axial, torsion, and impact loading. The mechanical properties of fiber-reinforced polymer composites are influenced by fiber, matrix, and interface. Carbon fibers are unique in their properties and their interaction with the polymer matrix, making carbon fiber-reinforced polymers stand out among other fiber-reinforced composites [22]. The properties of CFRP can be further enhanced by incorporating various additives.

The elastic modulus, often known as Young’s modulus, is a fundamental mechanical quantity that governs the stiffness or rigidity of a material. It assesses a material’s resistance to deformation when subjected to a load or stress. When analyzing composite mechanical properties, the elastic modulus value is an important variable to examine [23]. Carbon fiber’s smooth and chemically inert surface and its disordered graphite structure lead to inadequate interfacial performance between the fiber and resin due to its low surface energy. As a result, the usability of carbon fiber-reinforced polymer (CFRP) is limited [24]. Despite this, high-performance carbon fibers have been employed in producing composite materials for various applications such as aerospace, electronics, civil engineering, and sports. The interphase in these composites can enhance the compatibility between the fiber and matrix and the efficiency of load transfer to meet the demands of technological advancements [25].

Various methods have been employed to enhance the interface’s performance, which can be categorized into two groups. The first group involves modifying the surface shape of the fiber to increase the interphase area, while the second group involves altering the chemical groups on the surface to improve the chemical interaction with the matrix. However, changing the chemical groups is usually suitable for only one type of polymer, unlike expanding the interphase region by increasing the contact area with the fiber, which applies to every matrix [26]. The interface between the CF and the resin matrix largely influences the mechanical behavior of CF-reinforced epoxy (CF/EP) composites. Establishing a proper interface to facilitate efficient load transfer from the matrix to the reinforcements is important, which helps alleviate internal stress concentrations and significantly enhances the composites’ mechanical behavior and environmental stability [27]. The smooth and non-polar surface of CF makes it difficult for the resin matrix to wet and make contact, resulting in poor adhesion between the fiber and matrix. To improve the interfacial adhesion between CF and the matrix, various techniques have been employed to modify the CF surface. These techniques include sizing, coating, oxidation, chemical grafting, plasma treatment, electrophoretic deposition, and high-energy irradiation [28]. When examining interfacial adhesion, fiber-reinforced composites are typically viewed as having three primary components: the fibers, which bear the load; the backing material, which holds the fibers together and transfers the applied load between them; and the fiber-matrix interphase, which determines how well the fibers and matrix are bonded together [29]. In the last decade, extensive research has been conducted to improve the fiber-matrix interphase by treating the surfaces of CFs. Different methods have been used, including chemical grafting, electro-mechanical techniques, plasma treatment, radiation, and surface coating [30]. CB, a type of carbon material, has a microcrystalline structure with numerous functional groups on its surface. The carbon atoms on the surface of CB arrange themselves into hexagonal planes similar to graphite. Each layer of graphite in the CB crystallite has an ordered arrangement of carbon atoms, but the arrangement of carbon atoms in the surrounding layers is disordered and often referred to as quasi-graphite crystals [31]. During the manufacturing process of CB, which always occurs at high temperatures, the primary structure is formed as adjacent particles simultaneously melt together and occupy three-dimensional space. The secondary structure, which is a looser arrangement, gradually forms through physical adsorption, van der Waals forces, or static from the primary structure. The primary framework is hard to destroy, while the secondary structure, also called the transitory structure, is more vulnerable to mechanical damage during processing [32,33]. Carbon fiber (CF)-reinforced polymer composites are becoming more prevalent in primary aircraft structures, but a major issue with these materials is the occurrence of “delamination,” which can compromise the safety of the structure. To address this issue, current research is focused on finding solutions. However, the recent delamination problem experienced by the Boeing 787 Dreamliner highlights the challenges associated with adopting new materials and manufacturing processes. As the use of composite materials becomes more common in the aviation industry, the need to identify and correct delamination becomes increasingly critical. Composite materials offer numerous advantages, yet the lack of experience in manufacturing them leads to the likelihood of such problems. As a result, detecting and addressing delamination in these materials becomes a critical challenge as airplane components evolve [34]. Several efforts have been made to enhance the material properties of the individual components of fiber-reinforced composites, aiming to fulfill industrial demands with high standards. To achieve optimal performance from fiber-reinforced composites, it is crucial to have a strongly adhesive interface between the reinforcing fibers and the polymer matrix, along with a well-planned combination of these two components. The interfacial characteristics between the fibers and matrix have been regulated to enhance adhesive strength. This helps to effectively transfer external loads from the matrix to the fiber reinforcements, resulting in better performance of structural composite components [35]. Adding a small amount of carbon nanotubes to a matrix has been proven to enhance the mechanical properties of composite materials to a great extent [36,37,38,39]. FRP (fiber-reinforced polymer composites) are often used in structural applications and are therefore exposed to various weather conditions and loads. Unfortunately, hydrothermal aging can cause deterioration in several mechanical properties of FRP, such as tensile strength, flexural strength, wear resistance, inter-laminar shear strength (ILSS), and DC electrical conductivity [40,41,42]. The degradation in characteristics is believed to be caused by changes in chemical structure, the dissolution of covalent bonds, and chain scission in the polymer matrix. These alterations can cause composites to fail prematurely by causing interfacial and interlaminar failure, matrix erosion, matrix burning, and matrix cracking [43,44]. Incorporating carbon nanotubes (CNTs) into carbon fiber-reinforced polymer (CFRP) can be achieved by adding CNTs to the polymer matrix. However, achieving a homogenous distribution of the nanotubes can be achieved difficult. The strong Van der Waal forces cause the CNTs to clump together in the polymer matrix, leading to the development of clusters [45]. One solution to this problem is to use chemical vapor deposition (CVD) to directly graft nanotubes onto the surface of a fiber, which helps to overcome the clustering issue [46,47,48,49,50]. Modern composite materials used in various applications require high through-thickness thermal conductivity (TTTC) as internal heat generated by these systems needs to be dissipated efficiently [51,52,53,54,55]. Additionally, it has been demonstrated that adding micro and short fibers to concrete mixtures increases their pre-crack resistance and that distributing the fibers randomly enhances the concrete’s qualities in all directions. They also looked into how elements such as paste and silica sand used as aggregate metakaolin (MK) and GF contents affected the compressive and flexural strengths of the materials. Fiber content tests were used to study the qualities of recently placed concrete, such as its workability [56]. For the design of medical implants and other structural scaffolds, it is crucial to model the mechanical properties of carbon nanocomposites depending on input variables such as the %weight of Carbon Nanotubes (CNT) inclusions. Due to variations in circumstances, manufacturing methods, and inconsistent reagent characteristics utilized across industries and laboratories, current constitutive models for the mechanical properties of nanocomposites may not forecast adequately. Additionally, the mechanical characteristics of the designed goods exist as a probabilistic range rather than being deterministic [57]. Nonetheless, conventional continuous fiber prepreg methods have limitations when it comes to fabricating components with complex shapes. Challenges arise from the presence of defects such as dry yarn and resin-rich patches, which result in intricate internal stress distribution during the curing process. These defects significantly affect the functionality of the components. To establish reliable connections or precise positioning with other components, it is often necessary to resort to machining techniques, including the creation of holes and notches, in engineering applications [58,59]. The automotive and aviation sectors have devoted considerable attention to the utilization of fiber-reinforced plastic (FRP) composites. Extensive research has been conducted to address the mechanical joining challenges associated with these materials [60]. Specifically, carbon fiber-reinforced polymers (CFRP) have garnered significant interest in the aircraft industry due to their lightweight nature, cost-effectiveness, and high specific strength, making them a crucial structural component [61]. Moreover, the increasing popularity of fiber-reinforced plastic (FRP) composites can be attributed to their advantages, such as low weight, corrosion resistance, and high specific strength and stiffness. Consequently, various industries, including automotive, aerospace, and marine, have explored the potential applications of FRP composites [62]. The mechanical performance of fiber composites is influenced by the composition of the cementitious matrix and the presence of fibers. Previous research has extensively studied the mechanical and microstructural characteristics of cementitious materials under elevated temperatures. In addition, to improve the testing process, response surface methodology (RSM) is utilized to establish a model that correlates multiple parameters with various indicators. By analyzing the RSM results, the interactions between different variables and their impact on achieving multi-objective material optimization can be understood.

Yeong-Min Baek et al. [63] determined the mechanical characteristics of the recycled fiber and compared them to those of neat fibers using the single-fiber tensile test. The surfaces of recycled and pristine carbon fibers were studied and compared using FE-SEM and dynamic contact angle measurements. The study’s purpose was to investigate the viability of industrial use of recovered CF and/or recycled PET in CFRC. Hui-Jin Um et al. [64] examined the mechanical characteristics of PET matrix-based carbon fiber (CF)/PET composites with varying PET contents according to cooling rate. Banafsheh Sadeghi et al. [65] discussed recent research in three product categories (concrete, nonwoven textiles, and yarns) generated from recycled PET fibers, demonstrating the significant potential of PET fibers for the future industry. Kirill Kirshanov et al. [66] conducted a comprehensive review on the one hand to systematize the known methods of processing PET and copolyesters, highlighting their advantages and disadvantages, and on the other hand to demonstrate what valuable membrane products could be obtained and in what areas of the economy they could be used. Among the different approaches to the treatment of PET waste, we believe that chemical procedures hold the most potential.

Concerning this study, the initiative will concentrate on recycling carbon fiber waste, particularly from aerospace businesses. To make carbon fiber more environmentally friendly and sustainable, a solution must be found to recover the 30% of carbon fiber that is lost during production. The main objective of this study is to develop a recycled composite by using PET waste as a solution to the growing demand for both materials. The recycled carbon fibers were then processed chemically and mechanically in order to examine the mechanical strength of the carbon fibers that come from the pyrolysis process for CFRP. Various samples with varying quantities of CF (10%, 20%, 30%, and 40%) were tested to determine their mechanical properties. The recycled composites are tested under tensile circumstances to further investigate the effect of waste carbon reinforcing on the properties of polymers. Mechanical morphology was also evaluated using scanning electron microscopy. Following data analysis, it was discovered that samples containing 20% CF had the greatest elastic modulus value of all the mixtures.

## 2. Materials and Methods

An experimental study was conducted to evaluate the mechanical properties and morphology of a composite material composed of CF and PET waste. “The two main carbon fiber recycling approaches are mechanical and chemical recycling. Chemical recycling involves the use of chemicals to break down plastics” and produce raw materials that can be used to create new plastic products. PET waste was collected from bottle waste, while the Cy-com^®^977-2-35/40-12KHTS-268 criteria apply to the prepregs waste that STRATA provides; it has a 35/40% resin content, a curing class of 180, and a fiber area weight of 268 g/m^2^. The thermal, mechanical, and physical properties of PET are listed in Table 1 and Table 2. The mechanical testing was performed using a Universal Testing Machine, while the mechanical morphology of the material samples was examined using SEM. Sample preparation involved undergoing mechanical and chemical cycling processes. The subsequent subsections describe the sample preparation procedure and other testing methods employed in the study. In the sample preparation phase, the CF and PET waste were combined using specific techniques to create the composite material. The mechanical and chemical cycling processes involved subjecting the samples to repeated mechanical loading and exposing them to certain chemical treatments to simulate real-world conditions and assess their durability. The Universal Testing Machine played a crucial role in evaluating the mechanical properties of the composite material [67,68]. It allowed for precise measurements of parameters such as tensile strength, elastic modulus, and yield strength. This information provides insights into the material’s structural integrity and its ability to withstand external forces. SEM was utilized to analyze the mechanical morphology of the composite material. This technique provided detailed images and information about the samples’ internal structure and surface characteristics. By examining the microstructure and fiber distribution, researchers gained a deeper understanding of how the CF and PET waste interacted and contributed to the overall mechanical performance of the composite. The combination of these testing methods allowed for a comprehensive assessment of the mechanical properties and morphology of the CF/PET waste composite material. The results obtained from this experimental investigation are valuable for understanding the material’s behavior, identifying areas for improvement, and guiding future research and development efforts in the field of composite materials.

The combination of utilizing PET waste and incorporating carbon fiber makes a significant contribution to waste reduction efforts through the development of a novel approach. This approach focuses on converting carbon fiber-reinforced polymers (CFRPs) into carbon fiber (CF) and creating a composite material that exhibits wide-ranging application potential across various industries, including aerospace, automotive, and construction. By leveraging PET waste as a valuable resource, this innovative approach addresses the environmental challenges posed by CFRP waste. Instead of disposing of or incinerating CFRPs, which can lead to pollution and waste accumulation, the emphasis is placed on recovering and recycling valuable carbon fibers. This not only reduces the volume of waste sent to landfills but also promotes sustainable resource utilization by repurposing carbon fibers that possess exceptional mechanical properties. The resulting composite material, enriched with carbon fiber, offers a multitude of benefits and enhanced performance characteristics. Its versatility allows for application in different sectors, such as aerospace, automotive, and construction. In the aerospace industry, the composite’s lightweight yet robust nature makes it ideal for aircraft components, leading to improved fuel efficiency and reduced emissions. In the automotive sector, composite materials contribute to weight reduction, resulting in enhanced vehicle performance and fuel economy. Furthermore, in the construction field, the composite material provides structural strength while minimizing the overall weight of structures, thereby optimizing construction processes and enabling innovative design possibilities. By employing PET waste and incorporating carbon fiber, waste reduction efforts are amplified, simultaneously creating a composite material that can be harnessed across various industries. This holistic approach aligns with sustainable practices, promoting resource efficiency and minimizing the environmental impact associated with waste generation and disposal.

### 2.1. Pyrolysis

**Figure 1:**
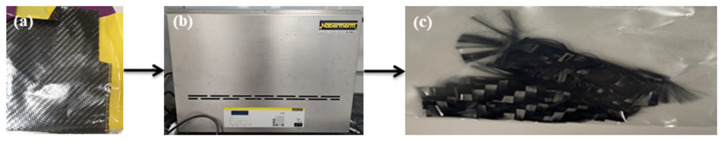
Schematic Diagram of pyrolysis process; (**a**) sample before pyrolysis machine (**b**) Pyrolysis machine (**c**) Sample after pyrolysis process.

A furnace (Nabertherm, Germany) is utilized for conducting pyrolysis on the CFRP material, as illustrated in Figure 1. It comprises three primary parameters that require configuration. The first parameter (T) represents the steady-state temperature at which the furnace operates during the pyrolysis process. The second parameter, referred to as (t1), signifies the rise time, indicating the duration it takes for the furnace to reach the desired steady-state temperature. Lastly, the third parameter, designated as (t2), represents the steady-state time, which refers to the duration the set temperature (T) is maintained before the furnace begins to cool down [71]. Operating the furnace is straightforward, as the parameters are manually inputted into the control panel. Once the parameters are set, the furnace operates autonomously until the pyrolysis process is completed. The sample preparation involves cutting the material to an appropriate size and determining its weight. Subsequently, the sample is placed in a ceramic combustion boat. Care is taken to gently position the boat in the center of the furnace’s tube to prevent any spillage during the process. Once the manual configuration of the desired parameters is completed on the control panel, the furnace initiates the heating process.

The illustrated furnace offers a controlled setting to perform pyrolysis on CFRP material, enabling researchers to investigate its properties through transformation and analysis. The examination of the pyrolyzed sample resulting from this process offers significant and valuable insights into the changes that take place in the structure and composition during pyrolysis. This advancement in knowledge greatly enhances our understanding of the behavior exhibited by CFRP materials. As a result, we can apply this knowledge to inform and improve the development and optimization of CFRP-based applications across a wide range of industries [72].

A series of samples underwent experimentation with different parameters to identify the optimal time and temperature conditions required for pyrolysis. The primary objective was to achieve the complete elimination of the epoxy component while simultaneously preserving carbon fibers that are clean, soft, and unburned. Extensive investigations led to the revelation that the most advantageous fiber outcomes were attained by implementing a 60-min duration for both the ramp-up and steady-state phases, combined with a pyrolysis temperature set at 500 °C.

The pyrolysis process was fine-tuned to perfection under these particular circumstances, resulting in the desired outcomes for carbon fiber production. The accompanying diagram vividly illustrates the exceptional results obtained by employing optimized pyrolysis conditions, showcasing the attainment of top-notch carbon fibers with remarkable quality. This valuable data holds immense potential for steering forthcoming research endeavors and industrial applications related to CFRP materials. It offers valuable insights into the specific process parameters that are imperative for achieving superior properties in carbon fibers.

**Chemical Process**

Chemical recycling is a process that involves the use of various chemicals to break down plastics into their basic building blocks or monomers. These monomers can then be used as raw materials to create new plastic products. Unlike traditional mechanical recycling, which involves melting and reforming plastics, chemical recycling focuses on breaking down the polymer chains of plastics through various chemical processes. This allows for a wider range of plastics to be recycled, including those that are typically challenging to recycle mechanically, such as multi-layered or contaminated plastics. There are different methods of chemical recycling. The first process is pyrolysis, where the process involves heating plastics in the absence of oxygen, which breaks them down into smaller molecules. These smaller molecules can then be further processed and refined to obtain monomers or other valuable chemicals. On the other hand, depolymerization breaks down plastics into their constituent monomers using chemical reactions. By selectively breaking the polymer chains, the original monomers can be recovered and used to produce new plastics. Moreover, solvolysis involves the use of solvents to dissolve and break down plastics. This process typically utilizes heat and pressure to facilitate the chemical reactions, leading to the recovery of monomers. Another process is gasification, which converts plastics into syngas, a mixture of hydrogen and carbon monoxide. The syngas can then be used as feedstock for various chemical processes to produce new plastics or other chemicals. Chemical recycling offers several potential benefits. It enables the recycling of a wider range of plastics, including those that are difficult to mechanically recycle. It can also help reduce plastic waste and the demand for virgin plastic production, contributing to a more circular economy. However, it’s worth noting that chemical recycling technologies are still evolving, and there are challenges to address, such as cost-effectiveness, scalability, and environmental impact.

### 2.2. Milling Process

**Figure 2:**
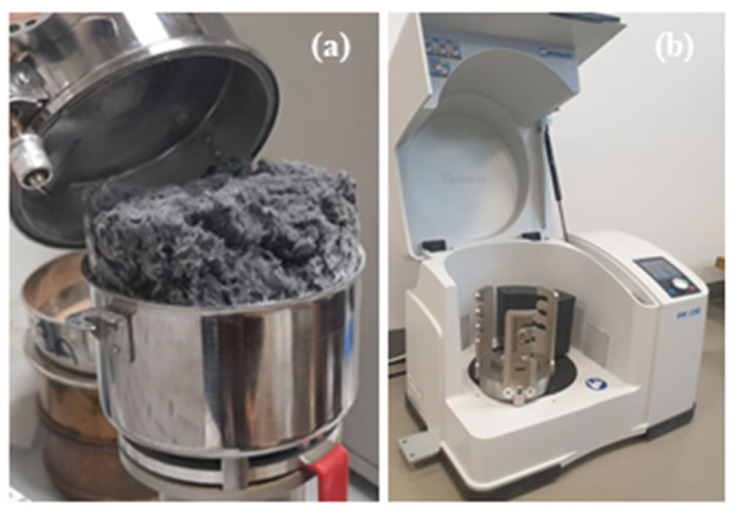
(**a**) Grinder mill machine and (**b**) Planetary Ball Mill PM 100.

Mechanical recycling techniques offer three options for processing CF materials: pre-pyrolysis, post-pyrolysis, or standalone mechanical recycling to obtain a powdered form that can be combined with other materials for future use (such as molding or mixing with other polymers). The choice of the appropriate grinding machine is essential to achieving the desired particle size and characteristics necessary for subsequent processing and utilization of the recycled CF. In order to determine the most suitable method for grinding CF, two machines were employed and compared: the Grinder Mill Machine and the Planetary Ball Mill PM 100 (PM-400, Retsch, Haan, Germany). These machines, depicted in Figure 2a,b, were utilized to assess their effectiveness in grinding CF materials.

The operation of this machine is relatively straightforward, making it user-friendly for grinding purposes, particularly for achieving fine particle sizes. Before usage, a one-day training session is typically conducted to familiarize users with the machine, its functionality, and safety precautions. Proper cleaning of the machine is essential before each operation, involving the use of a brush to eliminate any residual contaminants from previous use. In our specific case with CF, the material is initially cut into small pieces measuring approximately 5–10 mm. Subsequently, the cut material is loaded into the machine, and the machine cover is securely closed to initiate grinding. To achieve finer particle sizes, the machine’s running time can be increased until the desired particle size is attained. This machine provides a convenient and efficient solution for producing finely ground particles of CF for further processing and application in various industries.

### 2.3. Planetary Ball Mill PM 100

This particular machine is specifically designed to achieve a high level of fineness, even at the nanoscale, making it ideal for applications requiring fine grinding. It offers the flexibility of both wet and dry grinding processes. The machine’s advanced energy and speed control mechanisms ensure repeatable results, meaning consistent outcomes can be achieved using the same parameters. Its operation is based on the principle of differential speed between the grinding jar and the balls inside, resulting in a combination of frictional and impact forces that generate substantial dynamic energies. For well-preserved CF stored at low temperatures and remaining flexible, grinding it at 500 rpm for 5 min did not yield the desired grinding effect; instead, the sample exhibited a dough-like consistency. The corresponding figure illustrates the appearance of the sample after grinding. To explore potential improvements, the experiment will be repeated with an extended grinding time.

**Figure 3:**
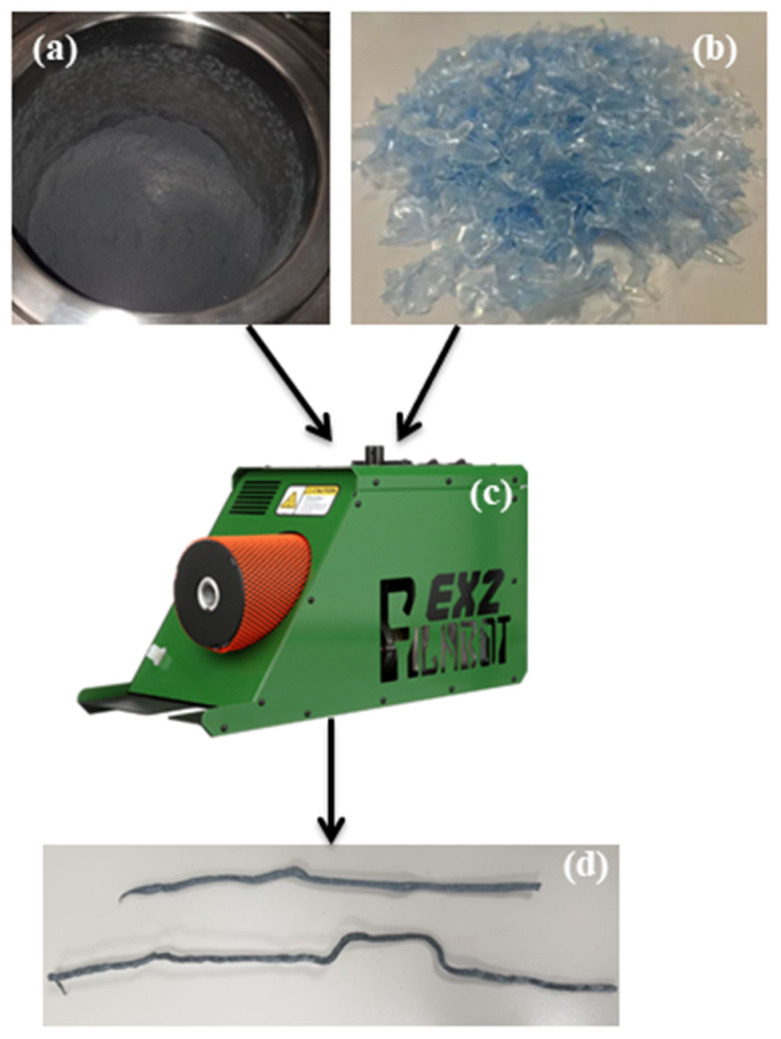
Material samples of (**a**) CF, (**b**) PET, (**c**) twin-screw extruder, and (**d**) prepared sample.

In contrast, CF that had been stored at room temperature showed more favorable results. After grinding it for 30 min at 450 rpm and subsequent sieving, the particle size was reduced to 20 µm. The corresponding figure showcases the sample following the grinding process. Additionally, Figure 3 presents a schematic diagram illustrating the layout of CF with PET waste in the composite material. There is a specific procedure to make composites from PET waste and carbon fiber. For simpler melting, the trash PET bottles were collected, cleaned, dried, and shredded. After melting, the fibers and fillers were added in the proper proportions and mixed until the mixture was uniform. The material was then poured into the prepared mold and pushed down to achieve even distribution [23].

### 2.4. Extrusion

After milling the Carbon Fiber sheets and turning them into powder, the next step is combining them with PET to form a material that can be used in industrial applications. Using only mechanical recycling alone to get a powder that is then mixed with PET at different percentages to test their strength and hardness. For this part, the extruder used to form the filaments was the Filabot EX2 extruder (Filabot, Barre VT, USA). This machine typically produces plastic filament for 3D printers and is relatively simple to operate. A one-day training session was held so that the student could learn how to use the equipment and the basic safety measures they should keep in mind when using it. To use the machine, first the materials, which in our case are CF/PET, have to be prepared. For the case of CF, they are prepared by cutting the sheets into small pieces (almost 5–10 mm) and milling them to get particles of a size range within the 50 µm range. The PET was obtained by cutting clean water bottles into small pieces with a size range of 5–10 mm. The pieces were then dried at a temperature of 160 °C for 4 h to remove any moisture. Finally, PET and milled CF are mixed to the required percentage, and the extruding process can be started using a jar. To start extrusion, the CF/PET mixture is filled into the opening at the top of the extruder. After that, the parameters, which are the extruding speed and temperature, are set, with a speed between 10 and 15 rpm and a temperature range between 245 °C and 248 °C. The process is then repeated several times with different CF percentages of 10%, 20%, 30%, 40%, and pure PET. The results obtained from this machine CF/PET mixture filaments of different CF percentages will then be tested to find the most strenuous and most challenging mixture.

### 2.5. Material Process

In order to assess the mechanical properties of the PET-CF samples obtained through extrusion, including tensile strength, yield strength, and ductility, a tensile test was conducted. This test aimed to compare the performance of the samples with different CF percentages to that of pure PET. To begin, the extruded samples were cut and carefully glued to create dog-bone-shaped specimens. This gluing process ensured that the samples were securely attached and ready for testing. Multiple specimens were prepared for each CF percentage, typically five in total.

**Figure 4:**
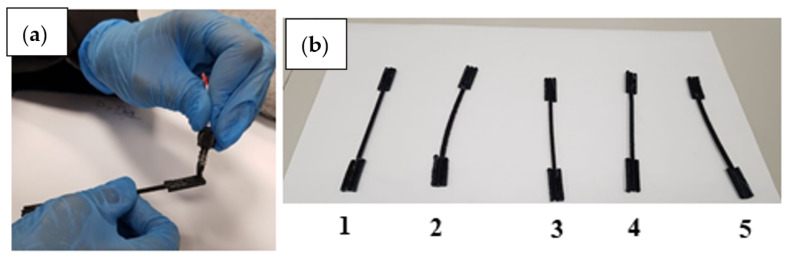
Sample Preparation (**a**) Bonding phase of the sample to make shoulders, (**b**) Finalized five tensile test samples.

Additionally, dog-bone samples made from pure PET before the extrusion process were prepared for testing. Before placing the samples in the testing machine, their dimensions were carefully measured. These measurements were necessary for subsequent calculations and analysis. The sample preparation procedure used for the tensile testing, including the gluing process, is illustrated in Figure 4.

**Figure 5:**
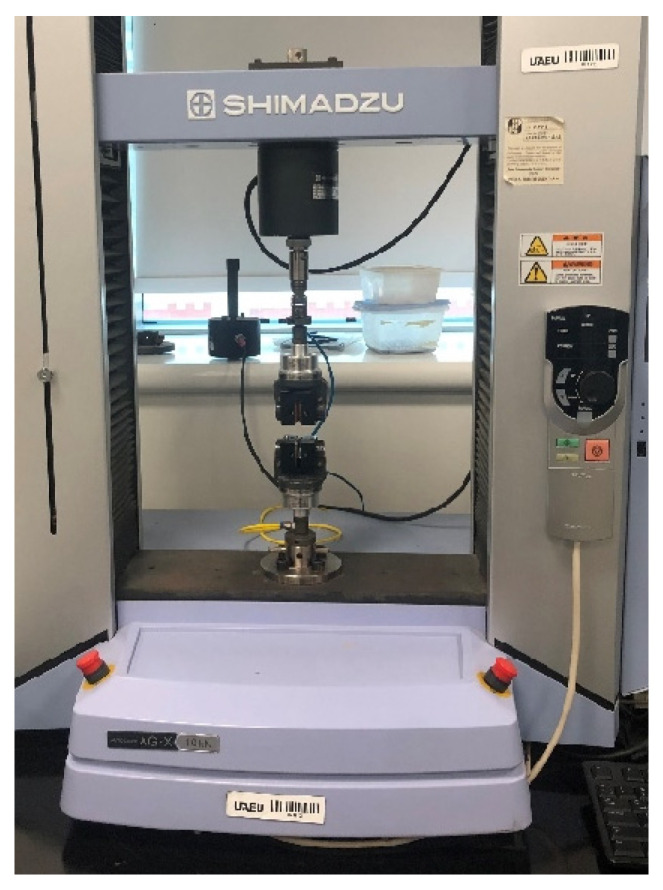
Tensile testing machine with a material sample.

Subsequently, the dog-bone-shaped material samples were securely positioned in the Tensile testing machine (UTM, Shimadzu, Kyoto, Japan), as depicted in Figure 5. The machine consists of various components that facilitate the testing process. The upper and lower grippers, designed to securely hold the material samples in place, ensure accurate and reliable measurements. A hydraulic press system is incorporated to apply forces in both tension and compression directions. The computer system is connected to a data acquisition system, enabling the collection and storage of data obtained from the machine. The Tensile testing machine yields various results that can be derived and analyzed according to the American Society for Testing and Materials standard (ASTM)-D 638. These results provide valuable insights into the mechanical behavior of the material under tension and compression.

## 3. Results

### 3.1. Mechanical Characteristics

In order to explore the various qualities, such as Scanning Electron Microscopy (SEM) analysis and tensile testing, CF/PET composite filaments have been produced from PET waste. CF is combined with PET waste CF shreds in a single extruder at (245–248 °C) and a speed of (10–15 rpm) to create CF/PET composite filaments. The amount of CF in the mixture affects the extruder’s temperature and speed. The typical filament diameter is 2 mm (the extruder nozzle has a 1.75 mm diameter). To create CF/PET composite filaments, different percentages of CF (10%, 20%, 30%, and 40%) were mixed with PET. To create CF/PET composite filaments blended with CF sheets and PET was employed. Many tensile specimens were punched from each sheet using a commercial dog-bone cutting machine. Due to its high resolution (0.001 mm) and precision (0.0002), the Mitutoyo thickness gauge (Model 547-526S) was used to measure the thickness of the thin layer. The average readings along the sample’s gauge length were considered in this investigation [16].

### 3.2. Mechanical Characterization

In order to evaluate the mechanical properties of the prepared composite samples derived from PET waste, a comprehensive experimental approach was employed. Tensile tests and a mechanical morphology analysis were conducted to thoroughly assess the mechanical strength and characterization of the composite samples. The composite samples were fabricated with different percentages of CF, namely 10%, 20%, 30%, and 40%. Various parameters were examined, including the elastic modulus, yield strength, tensile strength, toughness, and hardness. The experimental results were analyzed, and the computed values for these parameters were documented in Table 3, providing a comprehensive overview of the mechanical performance of the different material samples. The results obtained from the experimentation demonstrated that several crucial mechanical strength parameters, such as elastic modulus, yield strength, tensile strength, toughness, and hardness, were considered in evaluating the material samples. The elastic modulus, commonly known as Young’s modulus, is a fundamental mechanical parameter that determines a material’s stiffness or rigidity. It measures a material’s resistance to deformation under a load or stress. The elastic modulus value is an important quantity to consider while analyzing composite mechanical properties.

In terms of the various CF proportions evaluated, the composite with the highest elastic modulus value would normally be the one with the greatest concentration of carbon fibers. Carbon fibers have a substantially greater elastic modulus than the polymer matrix, which in CF composites is often epoxy resin. The proportion of stronger carbon fibers within the composite increases as the carbon fiber content increases. As a result, when carbon fiber concentrations grow, so does the composite’s overall stiffness or elastic modulus.

The evaluation of CF composites with carbon fiber concentrations of 10%, 20%, 30%, and 40% indicates that the composite with a 20% carbon fiber concentration is expected to exhibit the highest elastic modulus value. The higher carbon fiber content provides a stronger reinforcement effect, leading to improved stiffness and resistance to deformation. However, it is important to acknowledge that the behavior of composites can be influenced by various factors, including fiber orientation, matrix properties, and manufacturing techniques. Therefore, to determine the precise elastic modulus values for a specific CF composite system, it is necessary to conduct experimental testing and analysis.

It is important to note that the mechanical characterization of the composite samples played a vital role in understanding their structural integrity and performance. By assessing parameters such as elastic modulus, yield strength, tensile strength, toughness, and hardness, a comprehensive understanding of the mechanical strength of the material samples was achieved. The superiority of the composite sample containing 20% CF, as observed in terms of its greater strength, signifies the significant contribution of CF reinforcement to the mechanical properties of the composite. This reinforcing effect enhances strength and durability, making the composite sample with 20% CF a favorable choice in terms of mechanical performance. The results obtained from this mechanical characterization study provide valuable insights into the selection and optimization of composite formulations for PET waste-based materials. By understanding the mechanical behavior of the composites at different CF percentages, manufacturers and researchers can make informed decisions to develop composite materials with superior mechanical properties for specific applications.

**Figure 6:**
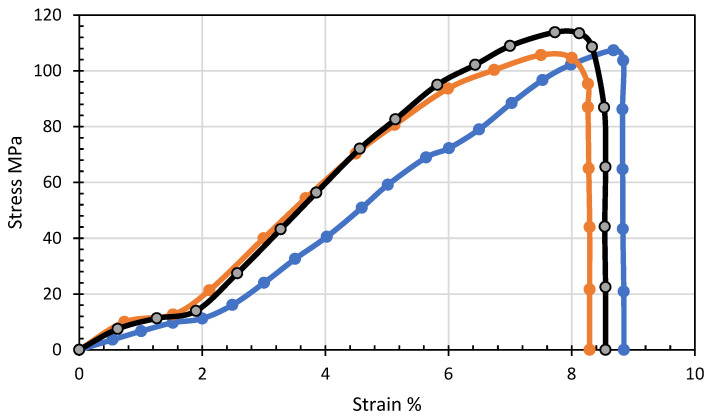
Stress-Strain curve for three samples of 10% PET composition.

**Figure 7:**
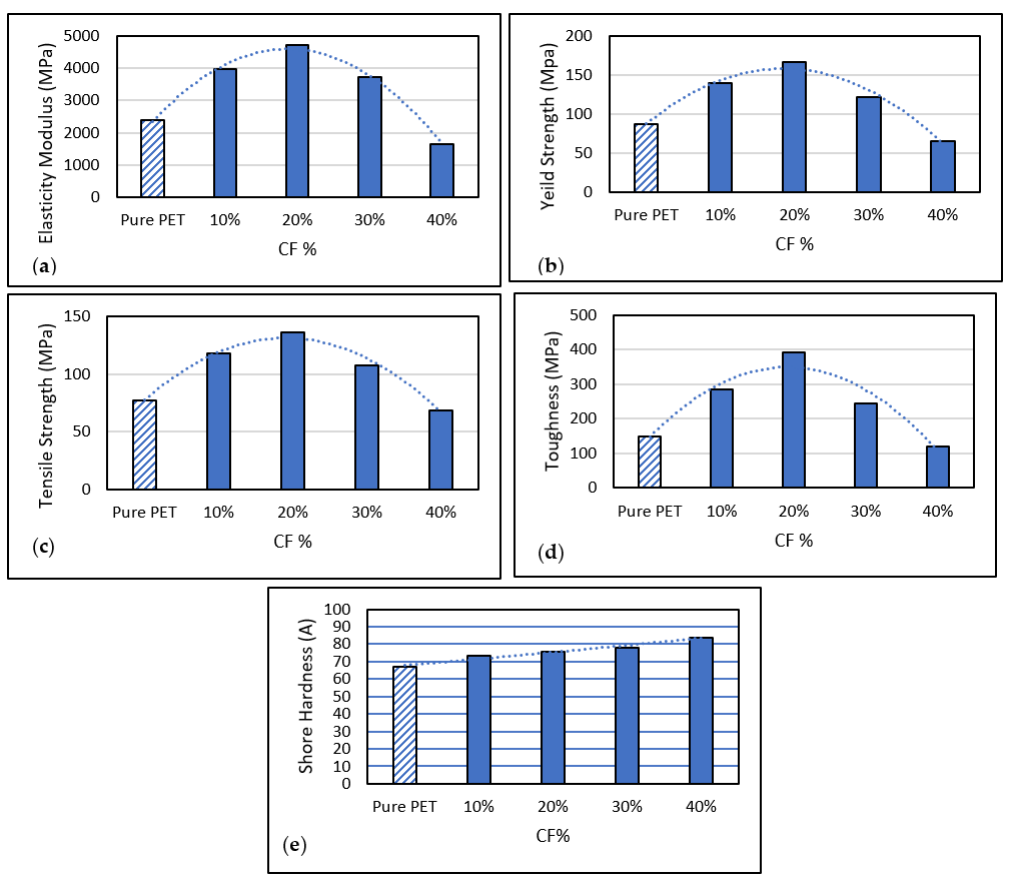
Graphical representations of different mechanical properties (**a**–**e**) at different CF content (%).

Figure 6 shows the stress-strain curve for a 10% PET composition. It is evident from the stress-strain curve that the maximum value of stress of 113.838 MPa was achieved at a strain of 7.72751. The analysis presented in Figure 7a provides insights into the elastic properties of the CF/PET composite. Among the various blends studied, the samples comprising 20% CF exhibited the highest elastic modulus value. This notable enhancement can be attributed to the reinforcing effect of the fibers. The elastic modulus of the filaments displayed consistent growth as the CF content increased until it reached its peak at 20%. Beyond this point, a decline in the elastic modulus was observed, corroborated by the graphical representations.

Based on the evaluation of carbon fiber (CF) composites with varying concentrations (10%, 20%, 30%, and 40%) of carbon fiber, it can be concluded that the composite containing 20% carbon fiber concentration is expected to exhibit the highest elastic modulus. The elastic modulus value reached a maximum of 4719.3 GPa. The primary reason for this notable increase in elastic modulus at the 20% carbon fiber concentration is attributed to the enhanced reinforcement effect, leading to improved stiffness and resistance to deformation. Consequently, the composite with a 20% carbon fiber concentration demonstrates superior mechanical properties compared to the other evaluated compositions. As the carbon fiber (CF) content in the filaments increased, the yield strength exhibited a rising trend until it reached its peak at a 20% CF concentration, beyond which it started to decline. The yield strength of the filaments containing 20% CF, as indicated by Figure 7b, measured 166.7 MPa, representing a significant improvement of 90.33% compared to pure recycled PET. The reinforcing properties of CF contributed to the increased yield strength. The addition of CF to recycled PET facilitated the formation of stronger and stiffer composites, leading to enhanced yield strength. The synergy between carbon fiber (CF) and the PET matrix drives the increased strength and stiffness observed in the composite material containing 20% CF. This interaction is accountable for the remarkable 90.33% higher elastic modulus compared to pure recycled PET. As a result of this significant improvement in mechanical properties, a broader range of applications, particularly those requiring elevated strength and stiffness, may become feasible [61]. Experimental results indicate that, among various mechanical properties, the elastic modulus exhibited the highest strength when the carbon fiber content was set at 20%. This finding highlights the significance of carbon fiber concentration in determining the composite material’s stiffness and resistance to deformation. The elastic modulus is a measure of a material’s ability to withstand applied forces without undergoing significant deformation. By incorporating a 20% carbon fiber content, the composite material achieves a remarkable increase in its elastic modulus, indicating improved rigidity and an enhanced ability to withstand external stresses. This suggests that the 20% carbon fiber concentration plays a critical role in maximizing the mechanical strength, particularly in terms of elastic modulus, of the composite material under investigation. Upon evaluating CF composites with varying carbon fiber concentrations of 10%, 20%, 30%, and 40%, it becomes evident that the composite containing a 20% carbon fiber concentration exhibits the highest elastic modulus value. In fact, the maximum recorded elastic modulus reaches an impressive 4719.3 GPa. This remarkable increase in elastic modulus at the 20% carbon fiber concentration can be attributed to the stronger reinforcement effect achieved by incorporating a higher amount of carbon fibers. Consequently, this reinforcement effect leads to a substantial enhancement in stiffness and deformation resistance. The greater elastic modulus observed in the composite with a 20% carbon fiber concentration highlights its exceptional ability to withstand external forces without significant deformation. This characteristic is of paramount importance in industries where mechanical strength and structural integrity are critical considerations. By effectively reinforcing the composite material with carbon fibers at a 20% concentration, a substantial improvement in its elastic modulus is achieved, showcasing its enhanced rigidity and resistance to deformation. This result underscores the importance of optimizing the carbon fiber concentration when designing CF composites for specific applications. By carefully tailoring the composition, it is possible to achieve superior mechanical properties, particularly in terms of elastic modulus. The reinforced composite with a 20% carbon fiber concentration not only demonstrates a significant increase in elastic modulus but also indicates its potential for delivering enhanced performance in terms of stiffness, durability, and resistance to deformation. So, the evaluation of CF composites reveals that the composite with a 20% carbon fiber concentration exhibits the highest elastic modulus value. The exceptional reinforcing effect achieved by the increased carbon fiber content leads to improved stiffness and deformation resistance. These findings emphasize the significance of carbon fiber concentration in optimizing the mechanical properties of CF composites for various industrial applications.

The outcome you stated indicates that adding carbon fiber (CF) increases the tensile strength of recycled polyethylene terephthalate (PET) up to a point, at which time adding more CF results in a drop in tensile strength. The Tensile Strength of the filaments increased as the percentage of CF increased until it reached its maximum at 20%, and after that, it started decreasing. Based on Figure 7c, the tensile strength of the 20% filament is 136.8 MPa, which is 76.2% greater than that of pure recycled PET. High-strength and high-stiffness materials like carbon fiber can reinforce polymers and enhance their Mechanical properties. Due to the reinforcement it offers, CF, when combined with recycled PET, results in a composite material with increased tensile strength. The characteristics of the materials involved can be used to explain this phenomenon. Beyond a certain extent, however, the amount of CF in the composite material might cause flaws or weaknesses in the substance, lowering its tensile strength. These flaws could include holes, gaps between the CF and PET matrix, or even breaks in the CF strands. In this instance, the maximum tensile strength is attained at 20% CF content, indicating that this is the ideal ratio for the specific type of recycled PET and CF employed in the study. Beyond this threshold, adding CF does not provide any more benefit in tensile strength and can harm the material’s overall mechanical qualities [62].

The toughness of the filaments exhibited an increasing trend as the percentage of CF increased until it reached its peak at 20%, after which it began to decrease. Figure 7d provides evidence of this behavior, with the 20% CF filament displaying a toughness value of 390.6 MPa, representing a substantial improvement of 163.5% compared to pure recycled PET. This enhancement in toughness can be attributed to the reinforcing effect of CF. Incorporating CF into the PET matrix contributes to the improved durability of the composite material. The increase in toughness with rising CF content is attributed to its ability to absorb energy and impede crack propagation. The synergistic interaction between CF and the PET matrix leads to the formation of a more robust and durable material. The remarkable improvement in toughness of 163.5% for the 20% CF composite, when compared to pure recycled PET, highlights the significant impact of CF reinforcement on the mechanical properties of the composite. This substantial enhancement in toughness expands the potential applications of the composite material, particularly in scenarios where high toughness, impact resistance, and resistance to various mechanical stresses are required [63].

The hardness of the filaments exhibited an upward trend as the percentage of CF increased, reaching its maximum value at 40% CF with a hardness of 83.8 Shore A. Figure 7e illustrates that the 20% CF filament displayed a hardness of 75.6 (Shore A), representing a 13% increase compared to pure recycled PET. It should be noted that the ideal CF ratio for achieving maximum hardness in the specific recycled PET and CF combination used in the study was found to be 40%. Going beyond this CF content may not further enhance hardness and could compromise other mechanical properties, such as toughness or ductility. The additional reinforcement provided by CF is primarily responsible for the 13% higher hardness observed in the 20% CF composite compared to pure recycled PET. This increased hardness renders the composite material suitable for applications that require high rigidity and hardness, such as those in the automotive, aerospace, or sporting goods industries. It is essential to acknowledge that various hardness testing methods, including Shore A, Shore D, and Rockwell hardness, each yield different hardness values. When comparing hardness values across different studies, it is crucial to consider the specific testing technique and conditions employed. Taking these factors into account ensures accurate and meaningful comparisons of hardness data [73]. The results obtained from this study have profound implications for the potential applications and performance of CF/PET composites in various industries. Through comprehensive analysis and experimentation, this study has yielded valuable insights into the behavior and characteristics of CF/PET composites, thereby serving as a crucial guide for further advancements and refinements in their design and manufacturing processes. One of the notable findings is the significant increase in the elastic modulus value observed at a 20% CF filament content. Compared to recycled CF, the elastic modulus experienced a substantial enhancement of 97.5%. This remarkable improvement can be attributed to the reinforcing effect of carbon fibers, which greatly enhance the stiffness and deformation resistance of the composites. The calculated elastic modulus value of 4719.3 MPa for CF with a 20% filament content exemplifies the impressive mechanical strength achieved through careful control of the carbon fiber concentration. These findings have far-reaching implications for the practical utilization of CF/PET composites in numerous applications. The enhanced elastic modulus values signify the superior structural integrity and rigidity of composites with a 20% CF filament content. This makes them particularly well-suited for applications that demand exceptional strength and performance. Industries such as aerospace, automotive, and construction can potentially benefit from these findings, as the improved mechanical properties of CF/PET composites can enable the development of lighter, stronger, and more durable materials for their specific requirements. Moreover, the insights gained from this study provide researchers, engineers, and manufacturers with valuable knowledge for optimizing the design and manufacturing processes of CF/PET composites. This knowledge can inform the selection of appropriate carbon fiber concentrations and guide the fabrication techniques to maximize the performance and functionality of the composites. Accordingly, the findings obtained from this study significantly enhance our understanding of the potential applications and performance of CF/PET composites. They provide valuable insights into the behavior and characteristics of these composites, facilitating further advancements and improvements in their design, manufacturing, and practical implementation.

### 3.3. SEM

Scanning Electron Microscopy (SEM) study is essential for understanding the structural and compositional properties of composite materials. It contains precise information about the composites’ surface morphology, topography, elemental composition, and interfacial characteristics.

The microscopic examination of CF-PET samples provided valuable information and observations that significantly contributed to understanding the characteristics of the composite material. Microscopic examination revealed the surface morphology of the CF-PET samples, offering insights into the arrangement, distribution, and orientation of carbon fibers within the PET matrix. This information is crucial for understanding the interfacial bonding between the fibers and the matrix as well as identifying any defects or irregularities present on the surface. Besides, the examination allowed for the assessment of fiber distribution throughout the composite. It provided information on whether the carbon fibers were evenly dispersed or clustered in specific regions, which impacts the mechanical properties and overall performance of the composite. Understanding fiber distribution helps optimize the manufacturing process and achieve desired material properties. The microscopic analysis provided a closer look at the interface between the carbon fibers and the PET matrix. It helped in assessing the quality of the fiber-matrix bonding, identifying any signs of debonding or delamination, and evaluating the effectiveness of the interface in transmitting stress between the components. These observations are crucial for enhancing interfacial adhesion and optimizing the composite’s mechanical properties. However, the process also contributed to understanding the interaction between the carbon fibers and the PET matrix. It provided insights into the wetting behavior of the matrix on the fiber surface, as well as any signs of chemical reactions or modifications at the interface. This knowledge is essential for improving the compatibility and load transfer between the filler and matrix, thereby enhancing the overall performance of the composite [74]. In order to analyze the morphology and composition of the extruded CF-PET samples in comparison to the extruded pure PET samples, a microscope was employed, and the elemental composition was characterized using JSM-6610 (JEOL PLUS/LA, Tokyo, Japan). All of the samples had to be placed on sticky carbon tape, which was then secured to the SEM stub before being coated with gold using a sputter coater while being exposed to inert gas and low vacuum. The samples that had been covered in gold were then evaluated in a vacuum environment. To ensure optimal results, the samples underwent a polishing process using various sanding and polishing materials before microscopic examination. The resulting material morphology data for both 100% recycled PET and the 20% filament sample are presented in Table 4. The microscopy analysis, conducted using scanning electron microscopes at a magnification of 5 µm, revealed distinct differences between the 20% filament sample and the pure recycled material. These differences are evident in Table 4, highlighting the impact of incorporating a 20% CF content. The microscopy characterization provided valuable insights into the structural and compositional aspects of the materials under investigation.

The study’s results indicate that adding carbon fiber (CF) to recycled PET significantly enhances the mechanical properties of the composite material. The hardness of the composite material demonstrates an increasing trend with increasing CF content until it reaches its peak at 20%, after which it begins to decline. This phenomenon can be attributed to CF’s remarkable energy absorption capability, which effectively inhibits crack propagation and contributes to the overall strength and durability of the composite material. As a result, incorporating CF leads to the development of a stronger and more resilient material. Microscopic examination reveals a clear distinction in the morphology and composition between the 20% filament sample and the sample composed solely of recycled PET. The observable differences can be attributed to the inclusion of carbon fiber (CF) in the composite material, which introduces modifications to the structure and characteristics of the final product. Before the microscope analysis, the samples undergo a thorough preparation process involving sanding and polishing materials. This preparation step allows for a more detailed and comprehensive examination of the samples, enabling a closer inspection of their features and properties.

The results of the study suggest that composite materials combining carbon fiber (CF) and recycled PET possess characteristics suitable for applications demanding high toughness and impact resistance. The synergistic interplay between CF and the PET matrix produces a more robust and durable material exhibiting improved mechanical properties. However, further investigation is necessary to fully understand the potential applications and specific attributes of these materials. Additional research and experimentation will provide a more comprehensive understanding of their capabilities, allowing for informed decision-making regarding their practical uses. The findings obtained from this study greatly contribute to our understanding of the wide-ranging applications and performance potential of CF/PET composites in diverse industries. These findings offer valuable insights into the behavior of CF/PET composites, serving as a pivotal guide for further advancements and enhancements in their design and manufacturing processes. One particularly intriguing discovery is the substantial increase in elastic modulus observed at a 20% CF filament content. The elastic modulus value experienced a remarkable boost of 97.5% compared to recycled CF, showcasing the exceptional reinforcing effect of carbon fibers. The calculated elastic modulus value for CF with a 20% filament content reached an impressive 4719.3 MPa. These findings shed light on the significant improvement in mechanical properties that can be achieved by carefully controlling the carbon fiber content in CF/PET composites. The enhanced elastic modulus values demonstrate the superior stiffness and deformation resistance offered by composites with a 20% CF filament content, making them highly desirable for applications that require exceptional strength and structural integrity. With these newfound insights, researchers and manufacturers can optimize the design and manufacturing processes of CF/PET composites to harness their full potential across various industries. The knowledge gained from this study sets the stage for further advancements in composite materials, paving the way for innovative and high-performance applications that can leverage the remarkable properties of CF/PET composites. A microscope was employed to examine the morphology and composition of the extruded CF-PET samples compared to pure PET samples to gain further insights into the composite’s characteristics. This type of study holds significant value for engineers and material scientists, enabling them to evaluate the mechanical properties of materials through the utilization of PET waste.

## 4. Conclusions

To investigate the mechanical properties of CF/PET composite filaments derived from PET waste, a comprehensive analysis was conducted utilizing tensile testing and Scanning Electron Microscopy (SEM) analysis. The key parameters examined in this study included the elastic modulus, yield strength, tensile strength, toughness, and hardness. To understand the material’s behavior comprehensively, these metrics were determined across various CF percentages, specifically 10%, 20%, 30%, and 40%. The present study employed a systematic experimental approach to comprehensively analyze CF/PET composite filaments derived from PET waste. Through the combined utilization of tensile testing and SEM analysis, valuable insights were obtained regarding the manufactured composite samples’ mechanical properties and structural characteristics. Among the material samples examined, it is evident that pure PET with a 20% CF content exhibits the highest strength. This enhancement can be attributed to the reinforcing effect of the CF fibers integrated within the composite structure. The elastic modulus of the filaments displays an increasing trend as the CF content rises until it reaches its peak at 20%, after which it gradually declines. These findings significantly enhance our understanding of the potential applications and performance of CF/PET composites across various industries. The findings derived from this study provide valuable insights into the behavior of CF/PET composites, serving as a guide for further advancements and improvements in their design and manufacturing processes. Of particular interest, at a 20% CF filament content, the elastic modulus value experienced a substantial increase by 97.5% compared to recycled CF. The calculated value for CF with a 20% filament content reached 4719.3 MPa. A microscope was employed to examine the morphology and composition of the extruded CF-PET samples compared to pure PET samples to gain further insights into the composite’s characteristics. This type of study holds significant value for engineers and material scientists, enabling them to evaluate the mechanical properties of materials through the utilization of PET waste. The knowledge garnered from this research offers a foundation for the development of innovative materials with improved mechanical performance and sustainability. By utilizing PET waste and incorporating CF, the potential for creating composite materials with enhanced properties expands, providing opportunities for various engineering applications.

**Recommendations:** It is recommended to proceed with further experimental work, such as thermal conductivity, to determine the thermal characteristics of the recycled composite in comparison with PET and CF. Apart from the production process, the Multi FLOW INDEX (MFI) is also an important parameter that could be studied in the future that give the designer a better understanding of the flow of the polymer under the production process [73]. Daohong Zhang et al. [74] introduced a single-fiber microdroplet test approach for assessing interfacial shear strength in natural fiber composites. They evaluated the effect of relative humidity (RH) in composite manufacturing on the interfacial shear strength and flexural characteristics of flax/unsaturated polyester composites. The interfacial shear strength of the flax/unsaturated polyester composite began to fall quickly at 70% RH and decreased more than sixfold at 90% RH.

## Figures and Tables

**Table 1:** Physical and thermal properties of PET [69,70].

Parameters	Values
Density (g/cm^3^)	1.38–1.56
Specific heat capacity (J/kg·K)	1000–1350
Thermal conductivity (W/mK, 23 °C)	0.15–0.4
Mw (g/mol)	30,000–80,000
Mn (g/mol)	8775
Elements content (wt%)	62%C, 4%H, 34%O
Lower heating values	22 MJ/kg
Higher heating values	36 MJ/kg
Refractive index	1.58–1.64
Freezing resistance (°C)	−50
Usable max. Temperature (°C)	70
O_2_ permeability (%)	0.1–0.4
CO_2_ permeability (%)	0.46
Water absorption (%, after 24 h)	0.3–0.5
Tg (°C)	67–80
Tcc (°C)	115–140
∆Tcc (J/g)	12–34
Tc (°C)	194–205
∆Tc (J/g)	29–55
∆Tm (J/g)	35–50
Tm (°C)	248–250

**Table 2:** Mechanical properties of PET [69,70].

Parameters	Values
Storage modulus at 25 °C (MPa)	2000–4200
Storage modulus at 80 °C (MPa)	242
Tensile strength (MPa)	40–60
Young’s modulus (MPa)	1000–3500
Flexural strength (MPa)	55–100
Elongation at break (%)	19–46
Flexural modulus (MPa)	2000–3500
Impact strength (kJ/m^2^)	4.6
Hardness (Shore-A)	96

**Table 3:** Estimated experimental mechanical properties.

Material ID	Elastic Modulus (GPa)	Yield Strength (GPa)	Tensile Strength (GPa)	Toughness (GPa)	Hardness (GPa)
Pure PET	2389.9	87.6	77.6	148.2	66.9
CFRP 10%	3970.0	140.0	118.0	285.0	73.2
CFRP 20%	4719.3	166.7	136.8	390.6	75.6
CFRP 30%	3730.0	121.3	108.0	245.0	78.2
CFRP 40%	1650.0	65.0	69.0	118.0	83.8

**Table 4:** Representations of sample morphology using SEM.

Pure Recycled PET	20% CF Composite
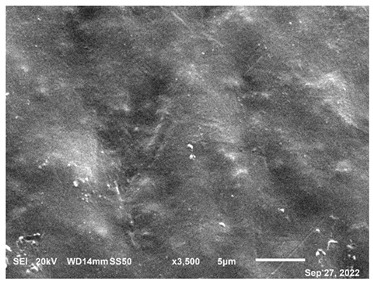	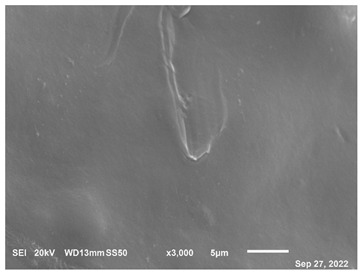
	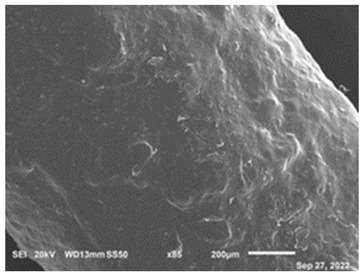

## Data Availability

The data presented in this study are available on request from the corresponding author.
